# Probiotic improves respiratory and gastrointestinal health, immune homeostasis, and gut microbiota composition in infants: a randomized controlled trial

**DOI:** 10.3389/fnut.2026.1746679

**Published:** 2026-03-09

**Authors:** Mageswaran Uma Mageswary, Lan Hanglian, Pin Li, Rocky Vester Richmond, Yusof Azianey, Joo Shun Tan, Min-Tze Liong, Adli Ali, Mishaleni Vejayantheran, Jiang Hua, He Jian, Intan Juliana Abd Hamid, Fahisham Taib, Yumei Zhang

**Affiliations:** 1 School of Industrial Technology, Universiti Sains Malaysia, Gelugor, Pulau Pinang, Malaysia; 2 National Technology Innovation Center for Dairy, Hohhot, China; 3 Department of Nutrition and Food Hygiene, School of Public Health, Peking University Health Science Center, Beijing, China; 4 Department of Pediatrics, Universiti Kebangsaan Malaysia Medical Centre, Faculty of Medicine, Universiti Kebangsaan Malaysia, Kuala Lumpur, Malaysia; 5 Kepala Batas Health Clinic, Kepala Batas, Pulau Pinang, Malaysia; 6 Laboratory of Microbiology, Immunology, and Metabolism, DiPROBIO (Shanghai) Co., Ltd, Shanghai, China; 7 Research Center, Hospital Tunku Ampuan Besar Tuanku Aishah Rohani, Universiti Kebangsaan Malaysia Specialist Children’s Hospital, Kuala Lumpur, Malaysia; 8 School of Nursery, Peking University Health Science Center, Beijing, China; 9 Primary Immunodeficiency Diseases Group, Department of Clinical Medicine, Pusat Kanser Tun Abdullah Ahmad Badawi, Universiti Sains Malaysia, Kepala Batas, Pulau Pinang, Malaysia; 10 Paediatric Department, School of Medical Sciences, Universiti Sains Malaysia, Kubang Kerian, Kelantan, Malaysia

**Keywords:** *Bifidobacterium infantis*, immune homeostasis, infant gut microbiota, mucosal immunity, probiotic, respiratory tract infections

## Abstract

**Introduction:**

The early postnatal period is a critical window for shaping the gut microbiota, which plays a pivotal role in immune maturation, infection resistance, and metabolic programming. Disruptions to this process may predispose infants to infections and allergic or metabolic disorders. Probiotics such as *Bifidobacterium infantis* have shown promise in modulating gut microbial ecology and immune function, but strain-specific and mechanistic evidence in infants remains limited. This study aimed to evaluate the effects of *B. infantis* YLGB-1496 supplementation on clinical outcomes, immune markers, and gut microbiota composition in healthy infants below one year of age.

**Methods:**

In a 12-week, randomized, double-blind, placebo-controlled trial, 119 healthy infants were enrolled (*B. infantis* YLGB-1496 *n*=59, placebo *n*=60). Participants received one daily sachet of *B. infantis* YLGB-1496 (1 × 10¹⁰ CFU) or placebo. Clinical outcomes for respiratory health and gastrointestinal (GI) health were assessed via validated questionnaires. Oral and fecal samples were collected for analysis of sIgA, cortisol, and cytokines (TNF-α, IFN-γ, IL-1β, IL-10, calprotectin). Gut microbiota was profiled by 16S rRNA sequencing, and diversity indices and taxonomic shifts were analyzed.

**Results:**

Compared with placebo, *B. infantis* YLGB-1496 supplementation was associated with consistent numerical reductions in respiratory symptom days, although these did not remain statistically significant after false discovery rate (FDR) adjustment. In contrast, gastrointestinal outcomes showed robust improvements after FDR correction, including reduced stomach ache (*q* = 0.010), lower diarrhea incidence (*q* < 0.001), and fewer diarrhea-related clinical visits (*q* < 0.001). Fecal sIgA remained elevated in the *B. infantis* YLGB-1496 group (*P* = 0.138 vs *P* = 0.000 in placebo), accompanied by increased IL-10 (*P* < 0.001) and reduced IL-1β (*P* = 0.002). Oral sIgA was enhanced (*P* = 0.001), while cortisol declined similarly in both groups. Microbiota analysis revealed enrichment of beneficial taxa in the *B. infantis* YLGB-1496 group with concurrent reductions in pathobionts. In contrast, the placebo group exhibited increases in Campylobacter, *Staphylococcus*, and *Desulfovibrio desulfuricans*, and decreases in *Faecalibacterium prausnitzii* and *Anaerostipes caccae*, indicative of dysbiosis. These compositional changes support improved gut barrier function and immune development.

**Clinical trial registration:**

https://clinicaltrials.gov/study/NCT05794815?term=NCT05794815&rank=1, Identifier: NCT05794815.

## 1 Introduction

The early postnatal period is a decisive phase for the establishment of the human gut microbiota, a dynamic microbial ecosystem that profoundly influences physiological, metabolic, and immune development. During this critical window, microbial colonization interacts continuously with host epithelial and immune maturation, contributing to nutrient metabolism, protection against pathogens, and education of immune tolerance mechanisms (1). The trajectory of this colonization process, particularly during the first year of life, has lifelong health implications. Deviations from normal microbial succession have been linked to heightened susceptibility to respiratory tract infections (RTI), allergies, metabolic disturbances, and immune-mediated diseases later in life (2).

Several factors can perturb this delicate microbial assembly, including delivery mode, antibiotic exposure, formula feeding, and environmental hygiene. Infants delivered via Caesarean section or exposed to antibiotics often exhibit reduced *Bifidobacterium* abundance, slower microbial maturation, and greater colonization by opportunistic taxa. These alterations correlate with impaired mucosal immunity and increased risk of infection. Consequently, interventions that can favorably modulate the gut microbiota during infancy have become an important focus of preventive health strategies.

Probiotics are defined as live microorganisms that confer a health benefit when administered in adequate amounts and represent a promising means of restoring microbial balance and supporting immune development (3). Among the diverse probiotic genera, *Bifidobacterium* species play a particularly crucial role in early life. These bacteria dominate the healthy infant gut, metabolize human milk oligosaccharides, and produce short-chain fatty acids (SCFAs) that reinforce epithelial integrity and regulate local immune responses (4). However, probiotic effects are highly strain-specific, and their clinical outcomes depend strongly on the host’s baseline microbiota composition, which is notably variable in infants (5).

While numerous studies have documented the clinical benefits of probiotics in reducing infectious diarrhea and antibiotic-associated dysbiosis in children (6, 7), the precise microbial mechanisms underlying these effects remain incompletely defined. Most investigations have focused on broad taxonomic changes at the genus level, overlooking specific compositional shifts that may drive physiological improvements. Understanding which bacterial taxa are selectively enriched or suppressed by particular probiotic strains is essential to elucidate their mechanisms of action and guide the rational selection of effective probiotic interventions.

*Bifidobacterium infantis* is one of the earliest colonizers of the human gut and exhibits unique adaptations to the infant gastrointestinal environment (8). It efficiently metabolizes milk-derived glycans and produces acetate and lactate, metabolites that acidify the intestinal milieu, suppress pathogens, and promotes the growth of commensal anaerobes. Emerging evidence suggests that *B. infantis* also exerts immunomodulatory effects by promoting secretory IgA (sIgA) production and enhancing anti-inflammatory cytokines, such as IL-10 (9). Nevertheless, there remains a paucity of randomized controlled trials evaluating specific *B. infantis* strains in healthy infants under 1 year of age, particularly those linking clinical benefits to detailed microbiota changes and immunological markers.

The current study was therefore designed to comprehensively assess the effects of *Bifidobacterium infantis* YLGB-1496 on clinical outcomes, immune parameters, and gut microbiota composition in healthy infants aged below 1 year. The strain was isolated from the breast milk of a healthy mother (10). We hypothesized that *B. infantis* YLGB-1496 would reduce GI infection symptoms and RTI symptoms, balance mucosal immune markers, and promote beneficial gut microbes. Using clinical and microbiome analyses, we aimed to show how probiotics support microbial and immune maturation during early life, a critical period for lifelong health.

## 2 Materials and methods

### 2.1 Study design and ethics

This study was a randomized, double-blind, placebo-controlled clinical trial evaluating the effects of *B. infantis* YLGB-1496 on gut microbiota composition, immune biomarkers, and clinical health outcomes in infants under 1 year of age. The trial adhered to the principles of the Declaration of Helsinki and the International Council for Harmonisation Good Clinical Practice (ICH-GCP) guidelines. Ethical approval was obtained from the Universiti Kebangsaan Malaysia (UKM) Research Ethics Committee (Approval No. UKM/PPI/111/8/JEP-2023-074). The study was prospectively registered at ClinicalTrials.gov on 20 March 2023 (Identifier: NCT05794815). Written informed consent was obtained from all parents or legal guardians before enrolment.

### 2.2 Study population and randomization

Healthy infants aged below 1 year were recruited through the UKM campus community and affiliated outreach programs. Inclusion criteria were (i) current weight between the 20th and 80th percentile on the national growth chart; (ii) regular consumption of standard infant formula without probiotic fortification; and (iii) parental commitment to study compliance. Exclusion criteria included chronic diseases, congenital abnormalities, long-term medication (>6 months), maternal metabolic or chronic illnesses, recent antibiotic use (<2 weeks before intervention), intake of probiotic or prebiotic supplements (other than permitted galactooligosaccharide <2 g/100 g in formula), food allergies, participation in other clinical studies within 4 weeks, or known hypersensitivity to study products.

**Figure 1:**
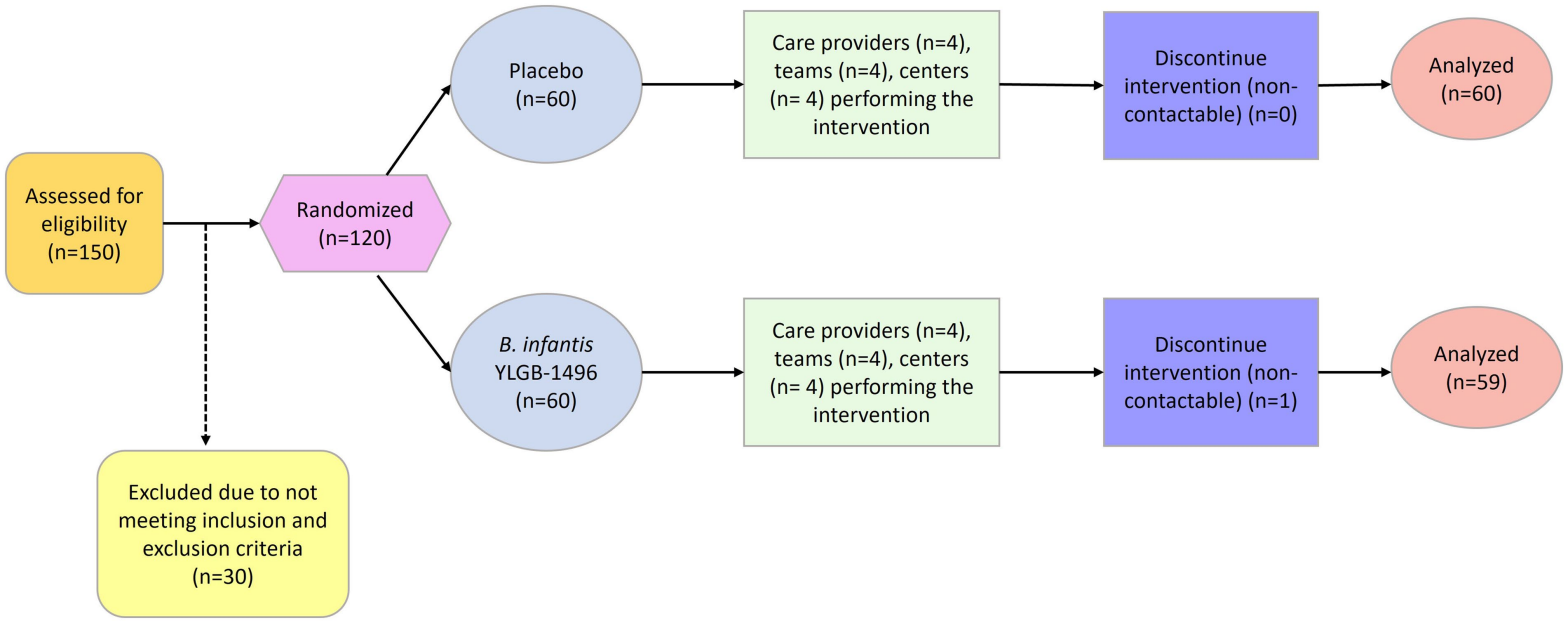
Flowchart shows the study enrollment, randomization, intervention, and follow-up processes. 150 infants screened, 120 were randomized equally into probiotic and placebo groups. One subject from the probiotic arm withdrew, resulting in 119 completing the study. No adverse events occurred. CONSORT diagram adapted to reflect participant disposition and analysis inclusion for the intention-to-treat cohort.

Randomization was computer-generated in a 1:1 ratio (probiotic vs. placebo) by an independent statistician with no contact with participants. Allocation was concealed until study completion. Both investigators and participants were blinded to treatment assignment. A total of 150 infants were screened, 120 met eligibility criteria and were randomized (*B. infantis* YLGB-1496 *n* = 60, placebo *n* = 60). One participant in the *B. infantis* YLGB-1496 arm was lost to follow-up, resulting in 119 evaluable subjects (Figure 1).

### 2.3 Intervention

The probiotic product contained *B. infantis* YLGB-1496 (1 × 10^10^ CFU per sachet) formulated as a light-yellow powder with a carrier matrix identical to that of the placebo, which contained only the carrier. Both products were identical in appearance, odor, and packaging, and stored at temperatures below 25 °C. Infants received one sachet per day for 12 consecutive weeks. Parents were instructed to mix the powder with lukewarm water or milk immediately before consumption.

### 2.4 Sample size and power calculation

Sample size was estimated for a parallel-arm design comparing continuous outcomes between two independent groups with 95% power and *α* = 0.05. Based on prior probiotic trials showing reductions in RTI-related clinical visits (11), 55 subjects per group were required. Accounting for a 10% attrition rate, 120 subjects were targeted for recruitment (*n* = 60 per arm).

### 2.5 Data collection and questionnaires

Validated and Malay-translated questionnaires (12) were administered at baseline (week 0), week 6, and week 12 to record demographic data, feeding history, and health outcomes, such as frequency and severity of RTI and GI infection symptoms, diarrhea episodes, clinical visits, and antibiotic use. All questionnaires were completed by parents under supervision from trained study personnel.

### 2.6 Sample collection

Oral swabs and fecal samples were collected at week 0, 6, and 12. Oral swabs were taken from the inner cheek (left and right) and stored at −80 °C for subsequent measurement of cortisol and secretory IgA (sIgA). Parents collected stool samples using sterile tubes containing RNAlater™ solution (Thermo Fisher Scientific, Waltham, MA, USA) and glass beads, stored at −80 °C until analysis. These were used for cytokine quantification and microbiota profiling.

### 2.7 Immunological and biochemical analyses

The preparation of fecal samples involved homogenization in phosphate-buffered saline and centrifugation (3,000× rpm, 20 min, 4 °C). The supernatant from this process was used for analysis. Oral swab samples were eluted in PBS by vortexing for 30 min, followed by centrifugation to collect the eluent. Fecal concentrations of sIgA, calprotectin, tumor necrosis factor-*α* (TNF-α), interferon-*γ* (IFN-γ), interleukin (IL)-1β, and IL-10 were quantified using commercial enzyme-linked immunosorbent assay (ELISA) kits (Elabscience, Hubei, Wuhan, China) according to manufacturer protocols. Oral samples were assayed for cortisol and sIgA using the same platform. All measurements were performed in duplicate, and intra-assay variability <10% was accepted as quality control. The absorbance was measured at 450 nm using a microplate reader (Multiskan Go, Thermo Fisher Scientific, Waltham, MA, USA).

### 2.8 Microbiota profiling

DNA was extracted from fecal samples following established protocols (13) and quantified using a NanoDrop 2000 UV–Vis spectrophotometer (Thermo Fisher Scientific, Waltham, MA, USA). The V3–V4 regions of the bacterial 16S rRNA gene were amplified using primers:

- 341F (5-CCTAYGGGRBGCASCAG-3)
- 806R (5-GGACTACHVGGGTWTCTAAT-3)

PCR reactions were performed in triplicate (20 μL volume) under the following cycling conditions: 95 °C for 3 min; 27 cycles of 95 °C for 30 s, 55 °C for 30 s, 72 °C for 45 s; and a final extension at 72 °C for 10 min. Amplicons were purified using the AxyPrep DNA Gel Extraction Kit (Axygen Biosciences, Corning, NY, USA), quantified using QuantiFluor™-ST (Promega Corporation, Madison, WI, USA), and sequenced (2 × 300 bp) on an Illumina MiSeq platform (Illumina, Inc., San Diego, CA, USA).

Raw FASTQ files were quality-filtered using Trimmomatic and merged by USEARCH v10.0. Operational taxonomic units (OTUs) were clustered at 97% sequence similarity using UPARSE. Taxonomic assignments were performed using the RDP Classifier against the SILVA 132 database (80% confidence threshold). Downstream analyses, including *α*- and *β*-diversity and differential abundance testing, were conducted in MicrobiomeAnalyst (phyloseq R package, v3.6.1) and STAMP v2.1.3.

### 2.9 Statistical analyses

All analyses followed an intention-to-treat approach using SPSS version 20.0 (IBM SPSS, Chicago, USA). Continuous variables were assessed using the Mann–Whitney *U* test due to non-parametric distribution; categorical data were compared with the chi-squared test. Within-group longitudinal comparisons were performed using the Wilcoxon signed-rank test. All tests were two-tailed with significance defined at *p* < 0.05. Data are reported as mean ± standard error unless otherwise specified.

## 3 Results

### 3.1 Participant flow and baseline characteristics

Of 150 infants screened, 120 met the eligibility criteria and were randomized equally to the *B. infantis* YLGB-1496 (*n* = 60) or placebo (*n* = 60) arms (Figure 1). One infant from the *B. infantis* YLGB-1496 group was lost to follow-up, leaving 119 participants (*B. infantis* YLGB-1496 *n* = 59; placebo *n* = 60) who completed the 12-week intervention. No adverse events or product-related complications were reported.

Baseline demographic and anthropometric characteristics were comparable between groups (Table 1). There were no significant differences in age, gender distribution, body weight, height, body mass index (BMI), or socioeconomic parameters (*p* > 0.05 for all). Lifestyle and environmental exposures, including household smoking, pet ownership, and dietary intake, were balanced between groups, indicating a homogenous study population. This ensured that observed outcomes were attributable to the intervention rather than pre-existing disparities.

**Table 1:** Baseline demographic and anthropometric characteristics of infants in the *B. infantis* YLGB-1496 (*n* = 59) and placebo (*n* = 60) groups.

Baseline Characteristics	Placebo	*B. infantis* YLGB-1496	*p*-value
Sample size (*n*)	60	59	
Gender, % (*n*)			
Male	52% (30)	37% (21)	0.115^*^
Female	48% (28)	63% (37)	
Age (months)	7.333 ± 0.408	8.119 ± 0.36	0.200
Body weight (kg)	7.795 ± 0.311	7.74 ± 0.252	0.645
Height (cm)	69.407 ± 1.381	68.33 ± 1.102	0.827
BMI	16.145 ± 0.369	16.28 ± 0.361	0.712
Smokers in family	0.3 ± 0.06	0.25 ± 0.057	0.579
Defecation frequency (per week)	9.775 ± 1.103	8.02 ± 0.813	0.390
History of food allergy	0.083 ± 0.036	0.12 ± 0.042	0.524
Antibiotic intake	0.033 ± 0.023	0.03 ± 0.024	0.986
Incidence of diarrhea for the past 12 months	0.408 ± 0.141	0.43 ± 0.14	0.552
Incidence of RTI for the past 12 months	0.117 ± 0.048	0.73 ± 0.412	0.085
Hospitalization for the past 12 months	0.35 ± 0.114	0.31 ± 0.081	0.723
Supplement intake	0.1 ± 0.039	0.17 ± 0.049	0.269
Dairy products consumption	0.333 ± 0.061	0.22 ± 0.054	0.170
Having pets at home	0.333 ± 0.061	0.22 ± 0.054	0.170

	% (*n*)	% (*n*)	*p*-value^*^
Family income status			
(a) Low (<RM6,000)	88 (53)	78 (46)	0.130
(b) High (>RM6,000)	12 (7)	22 (13)	
Number of people living together in a household			
(a) Less than or equal to 4 people	40 (24)	29 (17)	0.199
(b) More than 4 people	60 (36)	72 (42)	
House location			
(a) Urban	73 (44)	73 (43)	0.956
(b) Sub-urban	27 (16)	27 (16)	
Type of residence			
(a) Terrace/Storey/Bungalow	78 (47)	64 (38)	0.130
(b) Apartment	22 (13)	36 (21)	

*p*-value obtained via Mann–Whitney *U*-test; ^*^p-value obtained via chi-squared test. Values are presented as mean ± standard error or percentage (%). *p*-values denote between-group comparisons. BMI, body mass index.

No significant differences were observed between groups for any measured variables, indicating successful randomization and comparability at baseline.

### 3.2 Respiratory health outcomes

Respiratory health outcomes were evaluated longitudinally over 12 weeks, with between-group comparisons performed at weeks 0, 6, and 12. Continuous variables were analyzed using the Mann–Whitney *U* test and categorical variables using the chi-squared test. All *p*-values were adjusted for multiple comparisons using the Benjamini–Hochberg false discovery rate (FDR) procedure (Table 2).

**Table 2:** Number of days per subject experiencing respiratory-related symptoms in each study group (*n* = 119) randomly assigned to a double-blind administration with either placebo (*n* = 60) or *B. infantis* YLGB-1496 (*n* = 59).

Parameters	Week 0				Week 6				Week 12			
	Placebo	*B. infantis* YLGB-1496	*p-*value	*q-*value	Placebo	*B. infantis* YLGB-1496	*p-*value	*q-*value	Placebo	*B. infantis* YLGB-1496	*p-*value	*q-*value
Past month respiratory problems												
Yes (*n*, %)	12.20%	10.17%	0.668^*^	0.668	11.18%	6.10%	0.203^*^	0.305	10.17%	4.7%	0.094^*^	0.282
No (*n*, %)	48.80%	49.83%			49.82%	53.90%			50.83%	55.93%		
Clinical visit for respiratory problems in the past month												
Yes (*n*, %)	9.15%	8.14%	0.822^*^	0.976	6.10%	6.105	0.976^*^	0.976	8.13%	4.7%	0.235^*^	0.705
No (*n*, %)	51.85%	51.86%			54.90%	53.90%			50.83%	55.93%		
Antibiotic use for respiratory problems in the past month												
Yes (*n*, %)	5.8%	3.5%	0.479^*^	0.986	2.3%	2.3%	0.986^*^	0.986	4.7%	3.5%	0.714^*^	0.986
No (*n*, %)	55.92%	56.95%			58.96%	57.96%			56.93%	56.95%		
Number of days with respiratory symptoms (per week) in the past month												
Fever	0.35 ± 0.124	0.356 ± 0.126	0.969	0.969	0.517 ± 0.168	0.22 ± 0.088	0.498	0.560	0.5 ± 0.204	0.186 ± 0.1	0.473	0.559
Cough	0.858 ± 0.238	0.661 ± 0.228	0.623	0.652	0.992 ± 0.292	0.212 ± 0.137	0.023	0.149	0.867 ± 0.264	0.076 ± 0.043	0.031	0.149
Sneezing	0.825 ± 0.243	0.39 ± 0.149	0.202	0.293	0.625 ± 0.206	0.314 ± 0.17	0.118	0.252	0.933 ± 0.297	0.102 ± 0.05	0.069	0.208
Nose block	0.975 ± 0.287	0.576 ± 0.214	0.564	0.604	0.75 ± 0.258	0.263 ± 0.168	0.046	0.149	0.767 ± 0.232	0.161 ± 0.098	0.037	0.149
Wheezing	0.025 ± 0.025	0.203 ± 0.115	0.291	0.397	0.258 ± 0.138	0.144 ± 0.121	0.26	0.365	0.45 ± 0.182	0.051 ± 0.036	0.132	0.258
Sore throat	0.125 ± 0.054	0.068 ± 0.068	0.107	0.240	0.325 ± 0.152	0.144 ± 0.121	0.155	0.286	0.325 ± 0.176	0.051 ± 0.036	0.392	0.491
Runny nose	0.958 ± 0.268	0.466 ± 0.151	0.484	0.559	0.708 ± 0.22	0.339 ± 0.171	0.186	0.288	0.775 ± 0.243	0.229 ± 0.117	0.083	0.213
Poor appetite	0.7 ± 0.242	0.144 ± 0.079	0.08	0.213	0.408 ± 0.147	0.195 ± 0.085	0.524	0.575	0.625 ± 0.224	0.144 ± 0.079	0.129	0.258
Hoarseness	0.625 ± 0.242	0.28 ± 0.121	0.695	0.710	0.433 ± 0.173	0.119 ± 0.119	0.034	0.149	0.367 ± 0.162	0 ± 0	0.013	0.149
Body ache	0.025 ± 0.025	0 ± 0	0.321	0.425	0.283 ± 0.166	0.119 ± 0.119	0.183	0.288	0.417 ± 0.208	0 ± 0	0.045	0.149
Fatigue	0.492 ± 0.228	0.068 ± 0.068	0.095	0.226	0.208 ± 0.101	0.119 ± 0.076	0.478	0.559	0.367 ± 0.162	0 ± 0	0.013	0.149
Vomiting	0.542 ± 0.229	0.076 ± 0.043	0.176	0.288	0.308 ± 0.109	0.093 ± 0.072	0.032	0.149	0.172 ± 0.084	0 ± 0	0.022	0.149
Headache	0.05 ± 0.035	0 ± 0	0.159	0.286	0.258 ± 0.105	0.119 ± 0.076	0.2	0.293	0.267 ± 0.13	0 ± 0	0.045	0.149
Thick mucus	0.75 ± 0.242	0.415 ± 0.15	0.335	0.431	0.558 ± 0.198	0.144 ± 0.079	0.085	0.213	0.742 ± 0.248	0.051 ± 0.036	0.014	0.149
Pain swallowing	0.492 ± 0.228	0 ± 0	0.024	0.149	0.342 ± 0.134	0.068 ± 0.068	0.032	0.149	0.275 ± 0.149	0.025 ± 0.025	0.171	0.288

*p-*value obtained via Mann–Whitney *U*-test; ^*^p-value obtained via chi-squared test. Values represent mean ± standard error of days per participant per 12-week period. *q*-value were adjusted using the Benjamini–Hochberg false discovery rate (FDR) procedure.

At baseline, no statistically significant differences were observed between the placebo and *B. infantis* YLGB-1496 groups for any RTI outcome after FDR adjustment. Event-level outcomes, such as past-month RTI problems (*q* = 0.668), clinical visits for respiratory illness (*q* = 0.976), and antibiotic use (*q* = 0.986), were comparable between groups.

By Week 6, several respiratory symptoms showed numerically fewer symptomatic days in the *B. infantis* YLGB-1496 group, including cough (*q* = 0.149), nasal congestion (*q* = 0.149), hoarseness (*q* = 0.149), and vomiting (*q* = 0.149); however, none of these differences reached statistical significance after FDR adjustment. Similarly, event-level outcomes remained non-significant following correction, including past-month respiratory problems (*q* = 0.305), clinical visits (*q* = 0.976), and antibiotic use (*q* = 0.986).

At Week 12, the *B. infantis* YLGB-1496 group continued to demonstrate numerically lower symptom days for several respiratory complaints, including cough (*q* = 0.149), nasal congestion (*q* = 0.149), hoarseness (*q* = 0.149), body ache (*q* = 0.149), fatigue (*q* = 0.149), vomiting (*q* = 0.149), headache (*q* = 0.149), and thick mucus (*q* = 0.149). Nonetheless, all adjusted *p*-values exceeded the predefined FDR significance threshold, and no respiratory outcome demonstrated a statistically significant between-group difference after correction for multiple testing.

Overall, while directional reductions in respiratory symptom burden were observed in the *B. infantis* YLGB-1496 group over time, and these effects did not remain statistically significant after FDR adjustment, indicating that respiratory benefits were not robust when controlling for multiple comparisons.

### 3.3 Gastrointestinal health

Gastrointestinal (GI) outcomes were assessed using the same analytical approach, with *p*-values adjusted for multiple comparisons using the Benjamini–Hochberg FDR method (Table 3).

**Table 3:** Frequency of GI complaints per participant throughout the study (*n* = 119), randomly assigned to a double-blind administration with either placebo (*n* = 60) or *B. infantis* YLGB-1496 (*n* = 59).

Parameters	Week 0				Week 6				Week 12			
	Placebo	*B. infantis* YLGB-1496	*p-*value	*q-*value	Placebo	*B. infantis* YLGB-1496	*p-*value	*q-*value	Placebo	*B. infantis* YLGB-1496	*p-*value	*q-*value
Number of days with gastrointestinal symptoms (per week) in the past month												
Period (days) for stomach discomfort (such as flatulence and bloating) per week	0.450 ± 0.128	0.686 ± 0.163	0.158	0.194	1.608 ± 0.248	0.424 ± 0.104	0.001	0.002	0.625 ± 0.162	0.364 ± 0.114	0.336	0.386
Period (days) for stomach ache per week	0.267 ± 0.092	0.424 ± 0.138	0.332	0.386	1.333 ± 0.228	0.398 ± 0.102	0.007	0.011	0.850 ± 0.162	0.339 ± 0.112	0.005	0.010
Defecation times per week	10.283 ± 0.838	9.653 ± 0.735	0.858	0.891	9.633 ± 0.922	14.017 ± 1.343	0.028	0.042	10.833 ± 0.835	16.576 ± 1.202	0.001	0.002
Occurrence of diarrhea in the past month												
Yes (*n*, %)	20.33%	11.19%	0.068^*^	0.068	22.37%	8.14%	0.004^*^	0.006	23.38%	6.10%	<0.001^*^	<0.001
No (*n*, %)	40.66%	48.81%			38.63%	51.86%			37.62%	53.90%		
Past month diarrhea incident (number of times per child)	0.633 ± 0.139	0.458 ± 0.166	0.021	0.032	0.650 ± 0.116	0.305 ± 0.124	0.004	0.008	0.650 ± 0.114	0.203 ± 0.110	<0.001	<0.001
Past month clinical visit due to diarrhea incident (number of times per child)	0.467 ± 0.077	0.186 ± 0.051	0.005	0.009	0.383 ± 0.068	0.136 ± 0.045	0.004	0.007	0.517 ± 0.081	0.085 ± 0.037	<0.001	<0.001
Number of days with gastrointestinal symptoms (per week) in the past month												
Fever	0.800 ± 0.166	0.136 ± 0.070	0.001	0.002	0.708 ± 0.143	0.085 ± 0.061	<0.001	<0.001	1.108 ± 0.189	0.119 ± 0.073	<0.001	<0.001
Vomit	0.550 ± 0.115	0.051 ± 0.029	<0.001	0.001	0.55 ± 0.115	0.110 ± 0.079	0.001	0.002	0.242 ± 0.105	0.076 ± 0.076	0.06	0.083
Dysentry	0.00 ± 0.00	0.034 ± 0.024	0.152	0.191	0.00 ± 0.00	0.00 ± 0.00	1	1.000	0.00 ± 0.00	0.00 ± 0.00	1	1.000
Stomachache	0.342 ± 0.108	0.254 ± 0.089	0.507	0.570	0.925 ± 0.183	0.136 ± 0.066	0.001	0.002	1.117 ± 0.197	0.119 ± 0.064	<0.001	<0.001
Nausea	0.017 ± 0.017	0.034 ± 0.024	0.551	0.607	0.833 ± 0.173	0.034 ± 0.034	<0.001	<0.001	0.200 ± 0.097	0.00 ± 0.00	0.045	0.063
Loss of appetite	1.092 ± 0.209	0.119 ± 0.064	<0.001	0.001	0.933 ± 0.200	0.102 ± 0.052	<0.001	0.002	0.441 ± 0.173	0.034 ± 0.034	0.015	0.024
Fatigue	0.608 ± 0.148	0.119 ± 0.060	0.001	0.002	0.833 ± 0.173	0.051 ± 0.038	<0.001	<0.001	0.825 ± 0.163	0.051 ± 0.038	<0.001	<0.001
Dizziness	0.050 ± 0.037	0.017 ± 0.017	0.564	0.609	0.800 ± 0.173	0.00 ± 0.00	<0.001	<0.001	0.200 ± 0.115	0.00 ± 0.00	0.083	0.108
Headache	0.050 ± 0.037	0.034 ± 0.034	0.577	0.611	0.783 ± 0.170	0.00 ± 0.00	<0.001	<0.001	0.175 ± 0.101	0.00 ± 0.00	0.083	0.108
Dehydration	0.117 ± 0.068	0.153 ± 0.063	0.21	0.252	0.875 ± 0.183	0.034 ± 0.034	<0.001	<0.001	0.375 ± 0.146	0.00 ± 0.00	0.007	0.012
Rectal discomfort	0.050 ± 0.037	0.153 ± 0.063	0.084	0.108	0.283 ± 0.059	0.00 ± 0.00	<0.001	<0.001	0.183 ± 0.107	0.00 ± 0.00	0.045	0.063
Past month diarrhea incident (number of days)	0.800 ± 0.132	0.356 ± 0.107	0.006	0.010	0.783 ± 0.141	0.288 ± 0.114	0.003	0.007	1.233 ± 0.188	0.271 ± 0.120	<0.001	<0.001
Past month diarrhea incident (frequency per day)	1.350 ± 0.228	0.407 ± 0.121	0.002	0.003	1.350 ± 0.236	0.322 ± 0.122	0.001	0.003	1.333 ± 0.200	0.322 ± 0.140	<0.001	<0.001

*p-*value obtained via Mann–Whitney *U*-test; ^*^p-value obtained via Chi-square test. Values represent mean ± standard error of days per participant per 12-week period. *q*-values were adjusted using the Benjamini–Hochberg false discovery rate (FDR) procedure.

At baseline, GI infection symptom frequency and event-level outcomes were comparable between groups, with no significant differences observed after FDR adjustment. By Week 6, infants receiving *B. infantis* YLGB-1496 exhibited significant reductions in days with stomach discomfort (*q* = 0.002) and stomach ache (*q* = 0.011), alongside a significant increase in defecation frequency (*q* = 0.042). The incidence of diarrhea was significantly lower in the *B. infantis* YLGB-1496 group (*q* = 0.006), accompanied by fewer diarrhea episodes per child (*q* = 0.008) and fewer diarrhea-related clinical visits (*q* = 0.007).

By Week 12, these improvements were further strengthened. The *B. infantis* YLGB-1496 group reported significantly fewer days with stomach ache (*q* = 0.010), loss of appetite (*q* = 0.024), fatigue (*q* < 0.001), fever (*q* < 0.001), nausea (*q* = 0.063), dehydration (*q* = 0.012), and rectal discomfort (*q* = 0.063). Bowel regularity continued to improve, with higher defecation frequency (*q* = 0.002). Diarrhea outcomes remained strongly favorable, with lower incidence (*q* < 0.001), reduced episode frequency (*q* < 0.001), shorter duration (*q* < 0.001), and fewer clinical visits (*q* < 0.001).

Overall, gastrointestinal outcomes demonstrated consistent, clinically meaningful, and statistically robust improvements in the *B. infantis* YLGB-1496 group that persisted after correction for multiple testing.

### 3.4 Immune and stress biomarkers

#### 3.4.1 Fecal markers

**Figure 2:**
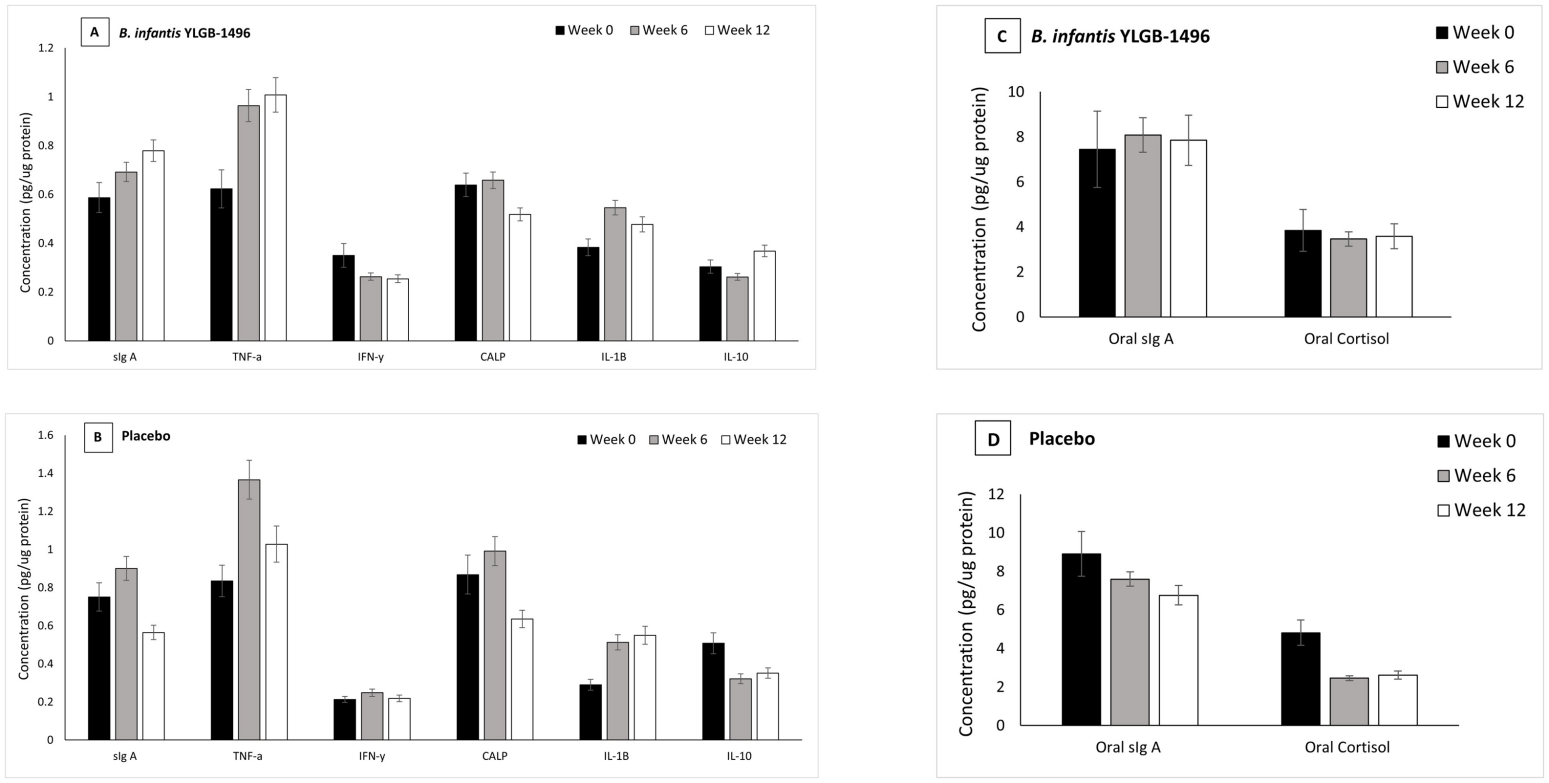
Fecal and oral immune and inflammatory biomarkers measured by ELISA in children receiving probiotic or placebo treatment over 12 weeks. **(A)** Fecal biomarkers (secretory IgA [sIgA], tumor necrosis factor-α [TNF-α], interferon-*γ* [IFN-γ], calprotectin [CALP], interleukin-1β [IL-1β], and interleukin-10 [IL-10]) in the *B. infantis* YLGB-1496 group. **(B)** Corresponding fecal biomarkers in the placebo group. **(C)** Oral sIgA and cortisol concentrations in the *B. infantis* YLGB-1496 group. **(D)** Oral sIgA and cortisol concentrations in the placebo group. Data are presented as mean ± SEM at Week 0, Week 6, and Week 12. Statistical analyses and exact *p*-values for all comparisons are provided in Table 4.

Distinct immunological trajectories were observed between groups (Table 4; Figures 2A,B). Both groups showed initial increases in fecal sIgA from baseline to Week 6 (placebo *p* = 0.003; *B. infantis* YLGB-1496 *p* = 0.002). Thereafter, sIgA levels declined sharply in the placebo group (*p* < 0.001) but remained elevated in the *B. infantis* YLGB-1496 arm (*p* = 0.138).

**Table 4:** Mean concentrations of fecal and oral immune and stress markers measured at baseline (Week 0), mid-intervention (Week 6), and post-intervention (Week 12) (*n* = 119) randomly assigned to a double-blind administration with either placebo (*n* = 60) or *B. infantis* YLGB-1496 (*n* = 59).

Protein markers	Week 0	Week 6	Week 12	*p-*value W0–W6^*^	*p-*value W6–W12^*^
Placebo					
Fecal					
Ig A	0.751 ± 0.074	0.9 ± 0.063	0.564 ± 0.038	0.003	<0.001
TNF-a	0.835 ± 0.083	1.366 ± 0.102	1.028 ± 0.095	<0.001	<0.001
IFN	0.213 ± 0.016	0.248 ± 0.019	0.218 ± 0.017	0.018	0.016
Calprotectin	0.868 ± 0.102	0.991 ± 0.076	0.635 ± 0.046	0.017	<0.001
IL-1B	0.29 ± 0.029	0.513 ± 0.04	0.549 ± 0.047	<0.001	0.193
IL-10	0.508 ± 0.055	0.321 ± 0.026	0.351 ± 0.027	<0.001	0.014
Oral					
Oral Ig A	8.902 ± 1.167	7.592 ± 0.372	6.76 ± 0.498	0.831	0.047
Oral Cortisol	4.816 ± 0.661	2.462 ± 0.124	2.614 ± 0.216	<0.001	0.797
*B. infantis* YLGB-1496					
Fecal					
Ig A	0.587 ± 0.061	0.692 ± 0.039	0.779 ± 0.044	0.002	0.138
TNF-a	0.623 ± 0.078	0.964 ± 0.066	1.008 ± 0.071	<0.001	0.824
IFN	0.35 ± 0.049	0.263 ± 0.015	0.254 ± 0.016	0.929	0.187
Calprotectin	0.639 ± 0.048	0.658 ± 0.034	0.518 ± 0.027	0.146	<0.001
IL-1B	0.383 ± 0.034	0.546 ± 0.03	0.477 ± 0.031	<0.001	0.002
IL-10	0.304 ± 0.027	0.262 ± 0.014	0.368 ± 0.024	0.284	<0.001
Oral					
Oral Ig A	7.448 ± 1.688	8.085 ± 0.764	7.849 ± 1.115	0.001	0.411
Oral Cortisol	3.842 ± 0.932	3.463 ± 0.312	3.582 ± 0.552	0.002	0.634

^*^p-value compared between week 0 with week 6 and week 6, with week 12 obtained via the Wilcoxon signed-rank test. Statistical test via Mann–Whitney *U*-test. Data are presented as mean ± standard deviation. *p* < 0.05 considered significant. sIgA, secretory immunoglobulin A; IL, interleukin; TNF, tumor necrosis factor; IFN, interferon gamma.

TNF-*α* increased in both groups up to Week 6 (*p* < 0.001) but declined in the placebo group thereafter (*p* < 0.001), while remaining stable in the *B. infantis* YLGB-1496 group (*p* = 0.824). IFN-*γ* fluctuated in the placebo group (rise to Week 6, *p* = 0.018; fall to Week 12, *p* = 0.016) but remained stable in the *B. infantis* YLGB-1496 arm (*p* > 0.05).

*B. infantis* YLGB-1496 supplementation maintained immune homeostasis with reduced volatility across pro-inflammatory markers. IL-10, a key anti-inflammatory cytokine, increased significantly between Weeks 6 and 12 in the *B. infantis* YLGB-1496 group (*p* < 0.001) but decreased in the placebo group (*p* = 0.014). Conversely, IL-1β and calprotectin levels declined significantly in the *B. infantis* YLGB-1496 arm by Week 12 (*p* = 0.002 and *p* < 0.001, respectively), further supporting an anti-inflammatory shift.

#### 3.4.2 Oral markers

Oral immune responses mirrored fecal patterns (Table 4; Figures 2C,D). Oral sIgA increased significantly from Week 0 to Week 6 in the *B. infantis* YLGB-1496 group (*p* = 0.001) and was maintained through Week 12 (*p* = 0.411), whereas the placebo group exhibited a reduction by Week 12 (*p* = 0.047). Oral cortisol levels decreased significantly from baseline to Week 6 in both groups (placebo *p* = 0.000; *B. infantis* YLGB-1496 *p* = 0.002) and remained stable thereafter, suggesting that the observed immune effects were not attributable to stress differences.

Overall, *B. infantis* YLGB-1496 elicited a balanced immune response characterized by sustained mucosal antibody defense (sIgA), reduced inflammatory volatility (IL-1β, TNF-*α*), and upregulated regulatory cytokines (IL-10), indicating improved mucosal immune equilibrium.

### 3.5 Alpha and Beta diversity of gut microbiota

**Figure 3:**
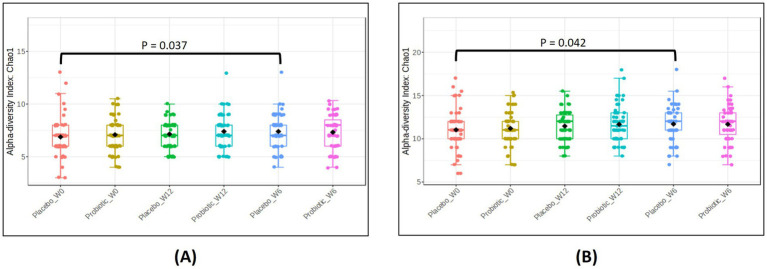
Alpha diversity plots for fecal microbiota at baseline (week-0), week-6 and end of study (week-12), upon administration of probiotic *B. infantis* YLGB-1496 or placebo. Differences in diversity within group as measured by the Chao1 index for **(A)** phylum, and **(B)** class. The line inside the box represents the median, whereas the whiskers represent the lowest and highest values within the interquartile range. Outliers, as well as individual sample values, are shown as dots. Statistical significance was analyzed using the Mann–Whitney *U* test. *n* = 119 (*B. infantis* YLGB-1496 *n* = 59 and placebo *n* = 60).

**Figure 4:**
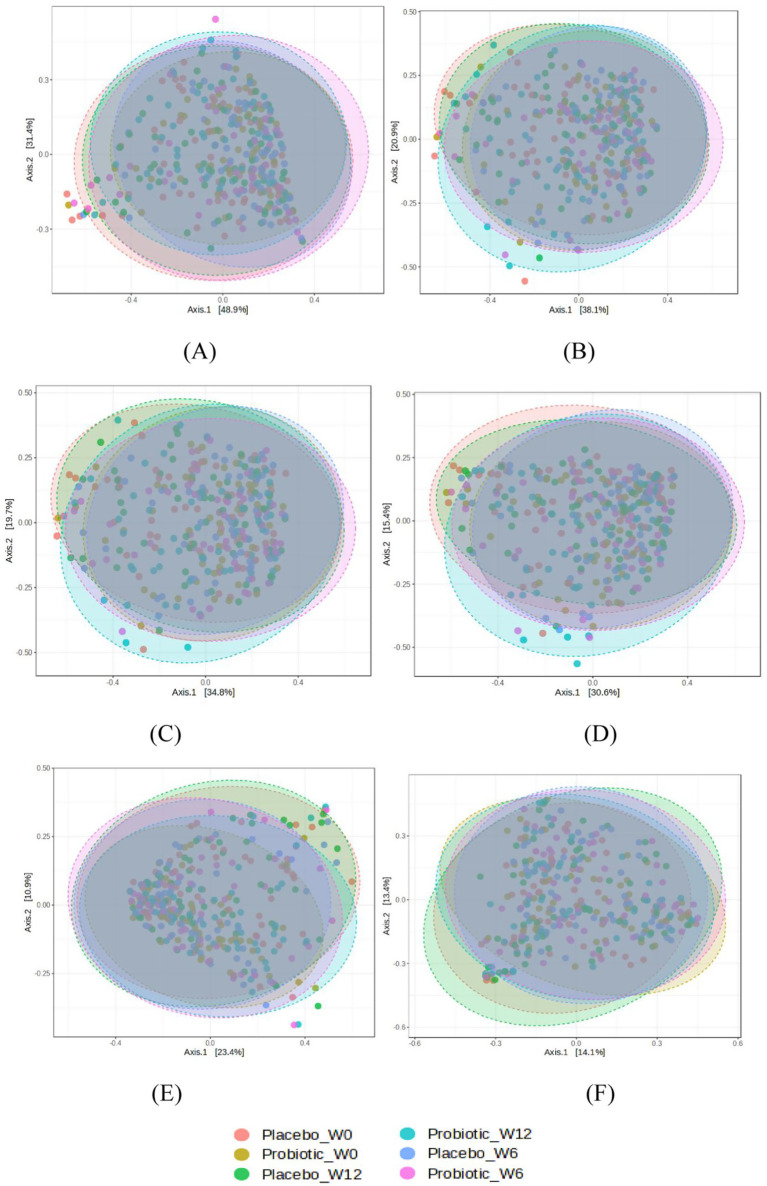
Principal coordinates analysis (PCoA) plots based on Bray–Curtis dissimilarity. Distinct baseline clustering between groups (PERMANOVA *p* < 0.05) converged by Week 12, indicating temporal stabilization of the microbiota community with probiotic use. Axes represent percentage of total variance explained by each principal coordinate. *p*-values determined by PERMANOVA test. *n* = 119 (*B. infantis* YLGB-1496 *n* = 59 and placebo *n* = 60). **(A)** phylum (*P* = 0.023), **(B)** class (*P* = 0.053), **(C)** order (*P* = 0.016), **(D)** family (*P* = 0.011), **(E)** genus (*P* = 0.009) and **(F)** species (*P* = 0.011).

Microbial diversity analyses revealed subtle but biologically relevant patterns (Figures 3, 4).

Alpha diversity, assessed using the Chao1 index, indicated that microbial richness increased at the phylum (Figure 3A) and class (Figure 3B) levels, transiently in the placebo group at Week 6 (*p* < 0.05) but returned to baseline by Week 12. No significant longitudinal changes were observed in the *B. infantis* YLGB-1496 group, suggesting that *B. infantis* YLGB-1496 supplementation did not alter overall within-sample diversity in healthy infants.

Beta diversity (Figure 4), measured using Bray–Curtis dissimilarity and visualized by PCoA, demonstrated distinct baseline microbial community structures between groups using PERMANOVA. Significant group differences were evident across multiple taxonomic ranks at Week 0 (A) phylum (*p* = 0.023), (B) class (*p* = 0.053), (C) order (*p* = 0.016), (D) family (*p* = 0.011), (E) genus (*p* = 0.009) and (F) species (*p* = 0.011) but not at Week 6 or 12, indicating convergence in community structure over time. This is consistent with previous reports that probiotics rarely induce major compositional shifts in healthy cohorts with resilient microbiota (14). These findings suggest that the probiotic exerted targeted, strain-specific effects on particular taxa rather than broad restructuring of microbial diversity.

### 3.6 Taxonomic compositional changes

**Figure 5:**
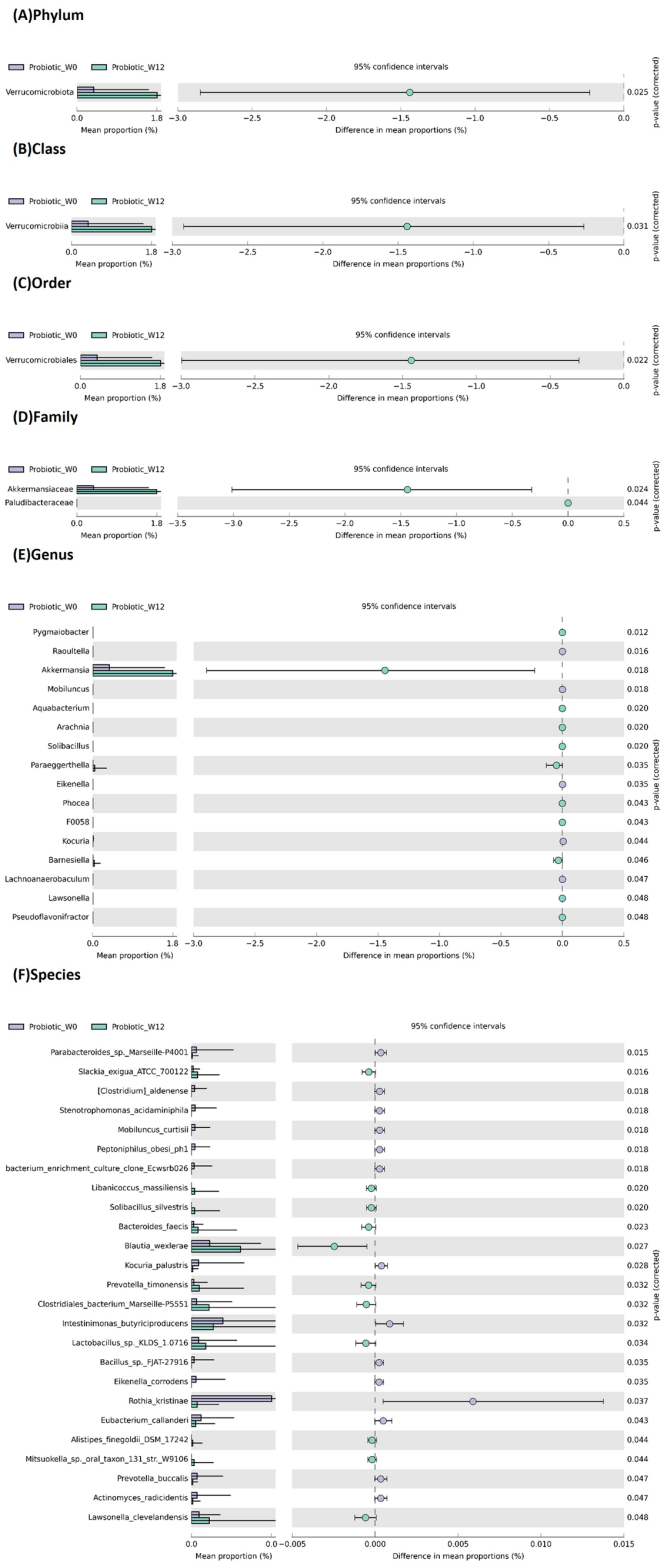
Extended bar plot showing average relative abundance across different taxonomic levels **(A)** phylum, **(B)** class, **(C)** order, **(D)** family, **(E)** genus and **(F)** species, following 12 weeks of supplementation of *B. infantis* YLGB-1496. Relative abundance determined by 16S rRNA gene sequencing; compositional differences tested by STAMP v2.1.3. False discovery rate (FDR) correction applied for multiple comparisons. Only taxa with *p* < 0.05 after FDR adjustment are shown *n* = 119 (*B. infantis* YLGB-1496 *n* = 59 and placebo *n* = 60).

**Figure 6:**
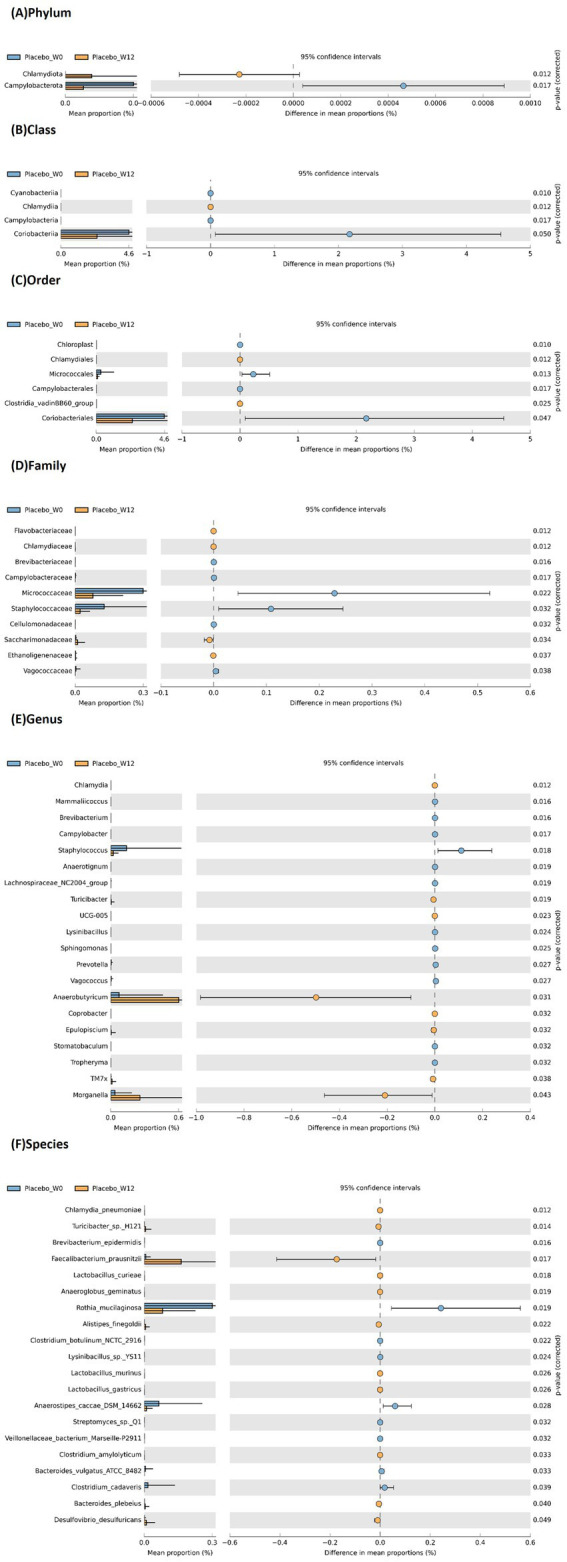
Extended bar plot showing average relative abundance across different taxonomic levels **(A)** phylum, **(B)** class, **(C)** order, **(D)** family, **(E)** genus and **(F)** species, following 12 weeks supplementation of placebo group. Relative abundance determined by 16S rRNA gene sequencing; compositional differences tested by STAMP v2.1.3. False discovery rate (FDR) correction applied for multiple comparisons. Only taxa with *p* < 0.05 after FDR adjustment are shown n = 119 (*B. infantis* YLGB-1496 *n* = 59 and placebo *n* = 60).

The 12-week *B. infantis* YLGB-1496 intervention induced distinct and significant shifts in the gut microbiota composition of infants compared to the placebo group. Comprehensive taxonomic profiling revealed distinct microbial signatures associated with *B. infantis* YLGB-1496 supplementation (Figures 5, 6).

In the *B. infantis* YLGB-1496 group (Figure 5), relative abundances increased for beneficial taxa across multiple ranks. These included the phylum Verrucomicrobiota (class Verrucomicrobiae; order Verrucomicrobiales; families Akkermansiaceae and Paludibacteraceae) and the genus *Akkermansia*. Additional enriched genera included *Pygmaiobacter*, *Aquabacterium*, *Arachnia*, *Solibacillus*, *Paraeggerthella*, *Phocea*, *Barnesiella*, *Lawsonella*, and *Pseudoflavonifractor*.

At the species level, significant increases were observed for *Slackia exigua* ATCC 700122, *Libanicoccus massiliensis*, *Solibacillus silvestris*, *Bacteroides faecis*, *Blautia wexlerae*, *Prevotella timonensis*, *Clostridiales bacterium Marseille* P5551, *Lactobacillus* sp. KLDS 1.0716, *Bacillus* sp. KLDS 1.0716, *Alistipes finegoldii* DSM 17242, and *Lawsonella clevelandensis*. Simultaneously, decreases occurred in potentially harmful taxa, such as *Raoultella*, *Mobiluncus*, *Eikenella*, *Kocuria*, *Lachnoanaerobaculum*, *Parabacteroides* sp. *Marseille* P4001, *Clostridium aldenense*, *Stenotrophomonas acidaminiphila*, *Mobiluncus curtisii*, *Peptoniphilus obesi* ph1, *Kocuria palustris*, *Intestimonas butyriciproducens*, *Bacillus* sp. FJAT 27916, *Eikenella corrodens*, *Rothia kristinae*, *Eubacterium callanderi*, *Prevotella buccalis*, and *Actinomyces radicidentis*.

In contrast, the placebo group (Figure 6) exhibited an increase in taxa associated with dysbiosis and inflammation, including the phylum Campylobacterota; classes Cyanobacteriia, Campylobacteriia, and Coriobacteriia; and families Campylobacteraceae, Staphylococcaceae, Brevibacteriaceae, Micrococcaceae, and Vagococcaceae. Genera that expanded included *Campylobacter*, *Staphylococcus*, *Mammaliicoccus*, *Vagococcus*, and *Tropheryma*. At the species level, increased abundance was noted for *Brevibacterium epidermis*, *Lactobacillus murinus*, *Lactobacillus gastricus*, *Clostridium amylolyticum*, *Bacteroides plebeius*, and *Desulfovibrio desulfuricans*.

Conversely, beneficial taxa declined in the placebo group, including *Chlamydia*, *Turicibacter*, UCG-005*, Anaerobutyricum*, *Coprobacter*, and *Faecalibacterium prausnitzii*. Loss of these SCFA producers and increases in pro-inflammatory taxa suggest a shift toward a less stable microbial environment.

Collectively, these findings demonstrate that *B. infantis* YLGB-1496 promoted a gut microbiota profile enriched in barrier-supporting and anti-inflammatory bacteria, whereas the placebo group displayed trends consistent with dysbiosis.

## 4 Discussion

This randomized, double-blind, placebo-controlled trial demonstrates that 12-week supplementation with *B. infantis* YLGB-1496 in healthy infants results in statistically robust improvements in gastrointestinal health with fewer diarrhea-related clinical visits and improved bowel regularity, while respiratory outcomes did not remain significant after false discovery rate correction. Immunologically, *B. infantis* YLGB-1496 stabilized mucosal sIgA levels, reduced inflammatory cytokine fluctuations, and enhanced IL-10 expression, indicating the establishment of a more balanced mucosal immune environment.

The present findings align with prior clinical trials demonstrating probiotic-mediated reductions in RTI and GI infection symptoms among children (7, 11). However, the current study extends previous work by integrating detailed immunological and microbiota profiling in infants under 1 year of age, a population with uniquely dynamic microbial development.

Although several respiratory symptoms exhibited numerical reductions in symptom days in the *B. infantis* YLGB-1496 group at Weeks 6 and 12, these effects did not withstand FDR adjustment and should therefore be interpreted cautiously. The absence of FDR-adjusted significance for respiratory event level outcomes, including infection incidence, clinical visits, and antibiotic use, suggests that the observed benefits of *B. infantis* YLGB-1496 are more strongly reflected in the modulation of symptom burden rather than in the prevention of respiratory infections during the study period. Notably, this pattern aligns with prior reports involving probiotic strains such as *Lactobacillus rhamnosus* and *Bifidobacterium longum*, which have demonstrated reductions in respiratory symptom duration (15–17), particularly cough and nasal congestion without corresponding changes in infection incidence, supporting the concept that probiotics may primarily influence symptom expression and mucosal resilience rather than infection acquisition.

In contrast, gastrointestinal outcomes demonstrated consistent, clinically meaningful, and statistically significant benefits after FDR correction. Reductions in diarrhea incidence, frequency, duration, and healthcare utilization, together with improvements in bowel regularity and GI-associated systemic symptoms, indicate a strong and biologically coherent effect of *B. infantis* YLGB-1496 on gut health. These findings support the role of *B. infantis* in maintaining intestinal homeostasis and reducing GI-related morbidity during early life. *B. infantis* YLGB-1496 reduced baseline symptoms and protected participants from further deterioration. Consistent improvements in symptom days, incidence, frequency, and clinical visits, all with *p* < 0.05, support the robustness of these findings despite reliance on self-reported data.

Taken together, the results suggest that the primary clinical benefit of *B. infantis* YLGB-1496 in this cohort is mediated through gastrointestinal pathways, with limited evidence for sustained respiratory benefit after correction for multiple comparisons. Future studies with larger sample sizes or respiratory-specific endpoints may be required to further clarify potential effects on respiratory health.

The immunological findings, specifically, maintenance of fecal sIgA and upregulation of IL-10, reflect mechanisms previously described in animal and human studies (18). IL-10 induction is a hallmark of immune regulation by *Bifidobacterium* species, contributing to anti-inflammatory effects in the GI tract. The sustained sIgA levels suggest enhanced mucosal protection, consistent with earlier reports linking probiotic use to reinforcement of epithelial immune defense and cross-mucosal immune communication. Additionally, the initial rise and later stabilization of TNF-*α* and IL-1β in the *B. infantis* YLGB-1496 group align with previous reports: certain *Lactobacillus* strains can trigger early pro-inflammatory signaling via macrophage activation, followed by a downregulation of cytokines such as TNF-α and IL-1β, reflecting a shift toward immune modulation and controlled inflammation (19–21). A stabilizing influence is further indicated by patterns in calprotectin, which is a recognized clinical marker for intestinal inflammation and permeability (22). The *B. infantis* YLGB-1496 group avoided the initial inflammatory spike in calprotectin seen in the placebo group, and both groups showed a beneficial decrease by Week 12. The systemic implications of gut-based immunomodulation are hinted at in the oral cavity data. The significant increase in oral IgA in the *B. infantis* YLGB-1496 group, contrasted with a decrease in the placebo group, points to a potential for gut-originating immune signals to influence distal mucosal sites, a phenomenon consistent with the concept of a common mucosal immune system (23).

Microbiota data align with and expand upon prior observations that *B. infantis* YLGB 1496 supplementation promotes the growth of beneficial taxa while suppressing opportunists. The most striking beneficial change in the *B. infantis* YLGB-1496 group was the marked increase in *Akkermansia* and its associated taxa (*Verrucomicrobiota* phylum, *Verrucomicrobiia* class). *Akkermansia muciniphila* is a well-known commensal bacterium renowned for strengthening the gut mucosal barrier by promoting mucin production (24). Its abundance is inversely correlated with inflammatory conditions and is considered a next-generation probiotic. The concurrent increase in other beneficial genera like *Blautia wexlerae* (a short-chain fatty acid (SCFA) producer) (25) and *Barnesiella* further supports an enhanced gut health phenotype. The decrease in potentially pathogenic or opportunistic genera in the *B. infantis* YLGB-1496 group, such as *Raoultella*, *Mobiluncus*, *Eikenella*, and *Stenotrophomonas*, suggested that the probiotic may have helped to suppress organisms associated with infections and dysbiosis (26).

In stark contrast, the placebo group displayed an increase in several taxa linked to dysbiosis and disease. The elevation of the entire *Campylobacterota* phylum, including the genus *Campylobacter*, is particularly concerning. *Campylobacter* sp. are established enteric pathogens, and their increased abundance, even in the absence of overt disease, may indicate a subclinical pro-inflammatory state (27). The rise in *Staphylococcus* and a *Clostridium* species (*C. amylolyticum*) further pointed toward a less stable microbial environment. The decrease in beneficial SCFA producers like *Faecalibacterium prausnitzii*, one of the most abundant and important butyrate-producing bacteria in the human gut with strong anti-inflammatory properties (28), and the depletion of *Anaerostipes caccae* likely indicate a significant loss of critical health-promoting, such as supporting gut epithelial integrity, immunoregulation, and microbial cross-feeding networks (29–31).

Collectively, these results support the role of *B. infantis* YLGB-1496 as a targeted probiotic strain capable of supporting gut barrier integrity, immune homeostasis, and systemic resilience in early life, a period crucial for long-term health programming.

### 4.1 Mechanistic insights

The observed improvements in respiratory and GI health outcomes are plausibly mediated by microbiota-driven immune modulation. The gut–mucosal axis is known to influence distal mucosal sites, including the respiratory tract, through shared immune pathways and cytokine signaling (32). The sustained elevation of fecal and oral sIgA in the *B. infantis* YLGB-1496 group suggests strengthened mucosal immunity extending beyond the intestine. sIgA acts as a first-line defense that neutralizes pathogens and toxins while preserving microbial diversity, thereby supporting tolerance and barrier protection (33).

The increase in IL-10 and reduction in IL-1β in the *B. infantis* YLGB-1496 arm provide biochemical evidence of immune homeostasis restoration. IL-10 suppresses NF-κB mediated inflammatory cascades (34), while IL-1β reduction reflects decreased epithelial stress and innate immune activation. These changes likely stem from enhanced interaction between commensal microbes and gut-associated lymphoid tissue (GALT), as has been documented in other *Bifidobacterium*-based interventions (18).

The increase in *Akkermansia* and *Blautia wexlerae* abundance offers further mechanistic insight. *Akkermansia muciniphila* enhances mucin layer turnover and epithelial tight-junction expression, improving gut barrier function and reducing endotoxin translocation (24). Meanwhile, *Blautia wexlerae* produces acetate and butyrate, key SCFAs that regulate inflammation, provide colonocyte energy, and modulate systemic immune tone (25). Together, these shifts likely promoted intestinal stability and anti-inflammatory signaling.

Conversely, the placebo group’s microbial shifts toward *Campylobacter*, *Staphylococcus*, and *Desulfovibrio* species suggest a drift toward low-grade inflammation and increased oxidative stress. *Desulfovibrio* species, for example, produce hydrogen sulfide, a toxic metabolite linked to mucosal irritation and epithelial injury (35). The concurrent loss of *Faecalibacterium prausnitzii*, a major butyrate producer with strong anti-inflammatory properties (28), further indicates compromised microbial resilience.

Taken together, these data suggest that *B. infantis* YLGB-1496 contributes to immune microbiota crosstalk that reinforces intestinal barrier integrity, enhances mucosal antibody production, and prevents dysbiotic transitions, thereby supporting both GI and respiratory health in early infancy.

### 4.2 Strengths and limitations

The major strength of this study lies in its comprehensive, multi-layered design, integrating clinical outcomes, immunological markers, and detailed microbiota analyses within a randomized, double-blind, placebo-controlled framework. The inclusion of both oral and fecal immune markers provides evidence for systemic mucosal effects, and the high retention rate (99%) ensures strong internal validity. Additionally, the use of 16S rRNA sequencing with taxonomic resolution to the species level offers nuanced insights into microbial modulation rarely achieved in infant probiotic studies.

Nonetheless, several limitations warrant consideration. First, the study population comprised healthy infants, which may have limited the magnitude of measurable benefit compared to diseased or high-risk cohorts. However, this focus allowed evaluation of probiotic effects under physiological conditions, and complementary evidence from our previous clinical trial in children with RTI symptoms supports the potential relevance of this strain in symptomatic populations. Second, dietary intake and environmental exposures were controlled by questionnaire but not objectively measured, which could introduce confounding. Future studies incorporating objective measures, such as detailed dietary records or environmental monitoring, would further strengthen exposure assessment. Third, although the 12-week intervention period was adequate to capture short-term microbiota and immune modulation, it does not provide insight into long-term microbial stability or sustained clinical outcomes following supplementation, highlighting the value of extended follow-up in future studies. Finally, although 16S rRNA profiling provides valuable compositional data, it does not capture functional metabolic activity; metagenomic or metabolomic analyses would strengthen mechanistic understanding.

Despite these limitations, the findings provide robust clinical and mechanistic evidence that *B. infantis* YLGB-1496 supports gut microbiota maturation, mucosal immunity, and systemic resilience during a critical developmental window. These results lay the groundwork for future longitudinal studies exploring persistent and functional outcomes of early-life probiotic supplementation. A key strength of this study is the integration of clinical outcomes, detailed immunological profiling, and gut microbiota analyses within a randomized, double-blind, placebo-controlled trial design. The exceptionally high retention rate (99%) minimizes attrition bias and strengthens internal validity. Furthermore, the inclusion of both oral and fecal immune markers provides a comprehensive assessment of mucosal immune responses. Microbiota profiling was conducted with taxonomic resolution to the species level, and statistical analyses incorporated false discovery rate correction to reduce the likelihood of type I error, ensuring robustness of microbiome findings.

## 5 Conclusion

This randomized, double-blind, placebo-controlled trial demonstrates that 12 weeks of *B. infantis* YLGB-1496 supplementation in healthy infants below 1 year of age significantly improves gastrointestinal health while supporting mucosal immune homeostasis and beneficial gut microbiota maturation. Infants receiving *B. infantis* YLGB-1496 showed robust improvements in diarrhea-related outcomes, sustained elevation of fecal and oral IgA, increased IL-10, and reductions in IL-1β and calprotectin, indicating a balanced anti-inflammatory immune profile. Although respiratory outcomes showed directional reductions in symptom burden, these did not remain statistically significant after correction for multiple comparisons. Microbiota analyses revealed enrichment of beneficial taxa and reductions in pathobionts. These findings collectively suggest that *B. infantis* YLGB-1496 exerts multifaceted benefits through microbiota-immune crosstalk, strengthening epithelial barrier integrity and promoting systemic immune stability. As early life may represent a critical window for immune and microbial development, such targeted probiotic interventions may offer a safe and effective approach to support infant health and resilience against infection and inflammation.

## Data Availability

The original contributions presented in the study are included in the article/supplementary material, further inquiries can be directed to the corresponding authors.
